# HPV-E7 Delivered by Engineered Exosomes Elicits a Protective CD8^+^ T Cell-Mediated Immune Response

**DOI:** 10.3390/v7031079

**Published:** 2015-03-09

**Authors:** Paola Di Bonito, Barbara Ridolfi, Sandra Columba-Cabezas, Andrea Giovannelli, Chiara Chiozzini, Francesco Manfredi, Simona Anticoli, Claudia Arenaccio, Maurizio Federico

**Affiliations:** 1 Department of Infectious, Parasitic and Immunomediated Diseases, Istituto Superiore di Sanità, 00161 Rome, Italy; E-Mails: paola.dibonito@iss.it (P.D.B.); andrea.giovannelli@iss.it (A.G.); 2 Department of Therapeutic Research and Medicine Evaluation, Istituto Superiore di Sanità, 00161 Rome, Italy; E-Mail: barbara.ridolfi@iss.it; 3 Department of Cell Biology and Neurosciences, Istituto Superiore di Sanità, 00161 Rome, Italy; E-Mail: sandra.columbacabezas@iss.it; 4 National AIDS Center, Istituto Superiore di Sanità, 00161 Rome, Italy; E-Mails: chiara.chiozzini@iss.it (C.C.); francesco.manfredi@iss.it (F.M.); simona.anticoli@iss.it (S.A.); claudia.arenaccio@guest.iss.it (C.A.); 5 Department of Science, University Roma Tre, 00146 Rome, Italy

**Keywords:** exosomes, CTL immunity, HIV-1 Nef, HPV-E7, antigen cross-presentation

## Abstract

We developed an innovative strategy to induce a cytotoxic T cell (CTL) immune response against protein antigens of choice. It relies on the production of exosomes, *i.e.*, nanovesicles spontaneously released by all cell types. We engineered the upload of huge amounts of protein antigens upon fusion with an anchoring protein (*i.e.*, HIV-1 Nef^mut^), which is an inactive protein incorporating in exosomes at high levels also when fused with foreign proteins. We compared the immunogenicity of engineered exosomes uploading human papillomavirus (HPV)-E7 with that of lentiviral virus-like particles (VLPs) incorporating equivalent amounts of the same antigen. These exosomes, whose limiting membrane was decorated with VSV-G, *i.e.*, an envelope protein inducing pH-dependent endosomal fusion, proved to be as immunogenic as the cognate VLPs. It is noteworthy that the immunogenicity of the engineered exosomes remained unaltered in the absence of VSV-G. Most important, we provide evidence that the inoculation in mouse of exosomes uploading HPV-E7 induces production of anti-HPV E7 CTLs, blocks the growth of syngeneic tumor cells inoculated after immunization, and controls the development of tumor cells inoculated before the exosome challenge. These results represent the proof-of-concept about both feasibility and efficacy of the Nef^mut^-based exosome platform for the induction of CD8^+^ T cell immunity.

## 1. Introduction

Eliciting a strong and broad cytotoxic T cell (CTL) immune response is expected to be of therapeutic relevance for treatment of several pathologies. We tried to establish a novel way to produce CD8^+^ T cell immunogens against protein antigens of choice. Our strategy was based on the use of engineered exosomes as immunogen carriers. Exosomes are vesicles of 50–100 nanometers forming intracellularly upon inward invagination of endosome membranes [1]. The intraluminal vesicles (ILVs) that are formed in this way become part of multivesicular bodies (MVBs). They are intracellular organelles consisting of a limiting membrane enclosing ILVs. MVBs can traffic either to lysosomes for degradation or to plasma membrane. In the latter case, MVBs release their vesicular contents in the extracellular milieu upon fusion with plasma membrane. Vesicles released by this mechanism are defined exosomes. Exosomes are part of the intercellular communication network [2]. They incorporate messenger RNAs, microRNAs, and proteins that are functional in target cells [3,4].

Exosomes are nanoparticles with a low intrinsic immunogenic profile. Their immunogenicity is essentially related to the amounts and quality of antigens they incorporate. Exosomes became the focus of many investigations aimed at testing their efficacy as anti-tumor immunostimulatory agents, and in some cases they reached approval for clinical trials [5,6,7]. Exosomes spontaneously uploading tumor antigens have been found to induce activation of specific anti-tumor T cell immunity [8,9]. These antigens are mainly *trans*-membrane proteins like gp100, TRP-1, Her2/neu, and CEA. Exosome-associated proteins can also affect the immune system in a non-antigen-specific way as in the case of immunosuppressive effects that are the consequence of either induction of apoptosis in T cells through the Fas-ligand pathway [10], differentiation of T_regs_ [11], or a decrease of cell cytotoxicity of natural killer cells [12]. On the other hand, exosomes can display unspecific immune-activation properties, most commonly as the result of association with molecular determinants inducing secretion of pro-inflammatory cytokines from target cells [13]. 

Tumor antigen-bearing exosomes have been tested in a number of clinical trials carried out on late-stage tumor patients. These trials demonstrated both feasibility and good tolerance to exosomes as cell-free vaccines in tumor patients. However, their therapeutic efficacy appeared quite limited. These results posed the need for new methods to increase the overall immunogenicity of therapeutic exosomes. This issue has been faced by engineering desired antigens to increase their association with exosomes. In this regard, two strategies have been described thus far. The first one exploits the binding of C1C2 domains of lactadherin to exosome lipids [14,15]. The other relies on coating exosomes with *Staphylococcus aureus* enterotoxin A tailed with a highly hydrophobic *trans*-membrane domain [16]. Both techniques result in a modification of the external contents of exosomes. 

The budding of human immunodeficiency virus (HIV) and related lenti- and retroviruses is preceded by the interaction with a number of cell factors also involved in exosome biogenesis, *i.e.*, Alix, Tsg101, and several other components of the endosomal sorting complex required for transport (ESCRT) [17]. HIV budding occurs at lipid rafts, *i.e.*, cell membrane microdomains enriched in cholesterol, phospholipids with saturated side chains, and sphingolipids. Also exosomal membranes contain lipid-raft microdomains [18]. The convergence of exosome and HIV biogenesis implies the possibility that viral products incorporate in exosomes. This was already proven for both Gag and Nef HIV-1 proteins. Nef associates with exosomes by anchoring its *N*-terminal myristoylation to lipid raft microdomains [19]. 

HIV-1 Nef is a 27 kDa protein lacking enzymatic activities [20]. It is the first HIV product synthesized in infected cells, thereby being expressed at levels comparable to those of HIV structural proteins. After synthesis at free ribosomes, Nef reaches both intracellular and plasma membranes to which it tightly interacts through both its *N*-terminal myristoylation and a stretch of basic amino acids located in alpha helix loop 1. Nef acts as a scaffold/adaptor element in triggering activation of signal transducing molecules. In most cases, this occurs upon Nef association with lipid raft microdomains. The fact that exosome membranes also are enriched in lipid raft microdomains explains why Nef can incorporate in both exosomes and HIV viral particles. 

We previously identified a ^V^153 ^L^ ^E^177^G^ Nef mutant incorporating at quite high levels in HIV-1 virions, HIV-1-based virus-like particles (VLPs) [21], and exosomes [22]. The incorporation efficiency of this mutant further increases by adding an *N*-terminal palmitoylation site through ^G^3^C^ mutation, expectedly leading to improved association with lipid rafts. This Nef mutant (referred to as Nef^mut^) is defective for basically all Nef functions [23], and its efficiency of incorporation in nanovesicles does not change significantly when foreign proteins are fused at its *C*-terminus [22].

Here, we provide evidence about both feasibility and efficacy of the Nef^mut^-based exosome system as a vehicle for inducing CD8^+^ T cell immunity. This strategy is expected to overcome the already proven limited CTL immunogenicity of exosomes, meanwhile maintaining their overall low basal immunogenicity and high biosafety profile. All these features, together with the demonstrated flexibility in terms of incorporation of foreign antigens and ease of production, make Nef^mut^-based exosomes a convenient candidate for a novel CTL vaccine platform.

## 2. Materials and Methods

### 2.1. Molecular Constructs

Both Nef^mut^ and Nef^mut^/HPV-E7 expressing vectors have been already described [24]. The Nef^mut^/MART-1 expressing vector has been recovered upon PCR amplification of MART-1 cDNA, and cloned in the previously described pTarget-Nef^mut^ shuttle vector [24]. The resulting molecular construct was checked for the absence of mutations. VSV-G was expressed by an IE-CMV-promoted vector.

### 2.2. Cell Cultures

HEK293T, 293/GPR37 [25], and TC-1 cells [26] were grown in Dulbecco’s modified Eagle’s medium plus 10% heat-inactivated fetal calf serum (FCS). Human monocytes were separated from peripheral blood mononuclear cells (PBMCs) using anti-CD14 microbeads (Miltenyi, Bergish Gladbach, Germany), and differentiated to iDCs upon 4–5 days of culture in Roswell Park Memorial Institute medium (RPMI) medium supplemented with 20% FCS, 30 ng/mL GM-CSF (Serotec Ltd., Kidlington, UK), and 500 units/mL IL-4 (R & D Systems, Minneapolis, MN, USA). The iDC phenotype was routinely characterized by fluorescence-activated cell sorting (FACS) analysis for the expression of CD11c and the absence of CD14 cell membrane markers. Both isolation and expansion of the CD8^+^ T cell clone specific for MART-1 have been previously described [27]. MART-1-specific CD8^+^ T cells recognize the HLA-A.02-restricted AAGIGILTV_27–35_ amino acid sequence. They were cultivated in RPMI plus 10% AB human serum (Gibco, Life Technologies, Monza, Italy), and regularly monitored for its specificity. Both mouse splenocytes and EL-4 cells, *i.e.*, murine thymic lymphoma CD4^+^ T cells originally obtained from C57 Bl/6 mice upon treatment with 9,10-dimethyl-1,2-benzanthracene [28], were cultivated in RPMI medium supplemented with 10% FCS.

### 2.3. VLP and Exosome Production

Lentiviral VLP preparations were obtained from supernatants of transfected 293/GPR37 cells. In these cells, HIV-1 *gag-pol* genes are expressed under control of an ecdysone-inducible promoter, so that the lentiviral particle production requires cell stimulation with the ecdysone analog ponasterone A. VLPs were obtained by transfecting Nef^mut^-based vectors in the presence or not of the vector expressing VSV-G by Lipofectamine 2000 (Invitrogen, Life Technologies, Monza, Italy). Transfected 293/GPR37 cells were induced 8 h post transfection with 5 mM sodium butyrate and 2 μM of ponasterone A. Twenty-four hours later, supernatants were replaced with fresh medium containing the inducers. VLP-containing supernatants were finally harvested both 24 and 48 h later, clarified, and concentrated by ultracentrifugation on 20% sucrose cushion at 100,000 × *g*, 2.5 h, 4 °C. VLP preparations were titrated in terms of HIV-1 CAp24 contents by quantitative ELISA (Innogenetics, Gent, Belgium).

To produce exosomes, HEK293T cells were transfected with vectors expressing the Nef^mut^-based fusion proteins. The cell cultures were washed 24 h later, reseeded in complete medium in the presence of exosome-deprived FCS, and the supernatants were harvested from 48 to 72 h after transfection. Exosomes were recovered through previously described methods [29]. In detail, supernatants were centrifuged at 500 × *g* for 10 min. Then, supernatants underwent differential centrifugations consisting in a first ultracentrifugation at 10,000 × *g* for 30 min. Supernatants were then harvested, filtered with 0.22 μM pore size, and ultracentrifuged at 70,000 × *g* for l h. Pelleted vesicles were resuspended in 1 × PBS, and ultracentrifuged again at 70,000 × *g* for 1 h. Afterwards, pellets were resuspended in 1:100 of the initial volume of 1 × PBS. The amounts of recovered exosomes were evaluated by measuring the activity of acetylcholinesterase (AchE), *i.e.*, a classical exosome marker [30], through the Amplex Red kit (Molecular Probes, Life Technologies, Monza, Italy). 

### 2.4. VLP and Exosome Characterization

Both VLP and exosome preparations were characterized by Western blot and FACS analyses. Through a previously described FACS-based assay for binding with cholera toxin, subunit B (CTX-B, Sigma-Aldrich, St. Louis, MO, USA) [22], we established that, in terms of number of nanovesicles, 1 μg HIV-1 CAp24 equivalent of VLPs equals 200 μU of AchE activity of exosomes [22,31]. Considering that an HIV-1 particle contains about 5,000 CAp24 molecules [32], one can calculate that 1 ng CAp24 of HIV-1 contains about 10^7^ vesicles. By consequence, we estimated the presence of about 10^10^ vesicles in both 1 μg CAp24 of HIV-1-based VLPs and 200 μU of AchE activity of exosomes. Equivalent amounts of nanovesicles were lysed in PBS, 1% Triton X-100 in the presence of anti-proteolytic agents, and then separated in 10% SDS-PAGE. Filters were revealed using the following Ab preparations: sheep anti-Nef antiserum ARP 444 (a generous gift of M. Harris, University of Leeds, Leeds, UK), anti-HPV E7 mAb from Zymed (Thermo Fisher, Waltham, MA, USA), and polyclonal anti-VSV-G protein from Immunology Consultant Laboratories (Portland, OR, USA). For semi-quantitative Western blot analysis, serial amounts of recombinant Nef obtained and quantified as previously described [33], were included as reference samples.

For the FACS analysis of nanovesicles, samples were incubated with 5 µL of surfactant-free white aldehyde/sulfate latex beads (Invitrogen, Life Technologies, Monza, Italy) overnight at r.t. on a rotating plate. Then, beads were incubated at 37 °C for 2 h with 1:50 diluted FITC-conjugated CTX-B. Therefore, bead–VLP complexes were treated with Cytoperm-Cytofix solution (BD Pharmingen, S. Diego, CA, USA) for 20 min at 4 °C, and finally labelled with 1:50 dilution of PE-conjugated KC57 anti-CAp24 mAb (Beckman-Coulter, Milano, Italy) 1 h at 4 °C. Bead-exosome complexes were labelled with PE-conjugated anti-CD63 mAb (BD Pharmingen) for 1 h at 4 °C. Finally, beads were washed, resuspended in 1*×* PBS-2% *v*/*v* formaldehyde, and FACS analyzed. 

### 2.5. Mice Immunization and Detection of IFN-γ Producing CD8^+^ T Lymphocytes

All studies with animals here described have been approved by the Ethical Committee of the Istituto Superiore di Sanità, Rome, Italy (protocol n. 555/SA/2012) according to Legislative Decree 116/92 which has implemented in Italy the European Directive 86/609/EEC on laboratory animal protection. Animals used in our research have been housed and treated according to the guidelines inserted in the aforementioned Legislative Decree. C57 Bl/6 mice were purchased from Charles River Laboratories (Calco, Italy), and inoculated subcutaneously (s.c.) 3 times at 2-week intervals with nanovesicles carrying equivalent amounts of antigens. Two weeks after the last inoculation, mice were sacrificed, and splenocytes put in culture in the presence of 5 μg/mL of 8- or 9-mer E7 peptides already identified to efficiently bind the H-2 K^b^ complex of C57 Bl/6 mice [34], *i.e.*, DLYCYEQL (aa 21–28), and RAHYNIVTF (aa 49–57). H-2 K^b^ binding HPV E6-specific KLPQLCTEL (aa. 18-26) and YDFAFRDL (aa 50–57) peptides [34] were used as control. After 4 days of incubation, IFN-γ Elispot assay was performed in triplicate conditions using commercially available reagents (Mabtech AB, Nacka Strand, Sweden). Spot-forming cells were analyzed and counted 16 h later using an Elispot reader (A.EL.VIS. Elispot reader and Analysis software GmbH Version 6.0, Hannover, Germany).

### 2.6. Cross-Presentation Assay

HLA-A.02 iDCs were challenged with equivalent amounts of exosomes (*i.e.*, 50 μU of AchE activity/10^5^ cells) associating or not with VSV-G. After 4 h of incubation, the iDCs were extensively washed and co-cultured in triplicate conditions at 1:2 ratio with the MART-1-specific HLA-A.02-restricted CD8^+^T cells in an IFN-γ Elispot microwell plate. After overnight incubation, IFN-γ Elispot assay was performed using commercially available reagents, and spot-forming cells were analyzed using an Elispot reader.

### 2.7. Detection of Anti-E7 Abs

Sera from inoculated mice were pooled, and two-fold serial dilutions starting from 1:100 were assayed for the presence of anti-E7 Abs. The end-point dilution corresponded to a <0.1 OD absorbance at 450 nm. Each serum was assayed in triplicate, and the mean of the absorbance value was taken as final readout. Recombinant E7 produced as described [35] was used for the assay. The protein was adsorbed overnight at 4 °C in carbonate buffer (pH 9.4) into Maxisorp microtiter plates (NUNC) at the concentrations of 0.25 µg/well. After a blocking step of 2 h of at 37 °C in PBS containing 3% non-fat dry milk (NFDM), plates were incubated at 37 °C for 1 h with 100 µL of serially diluted mouse sera in 1% NFDM-PBS. Specific antigen−antibody complexes were detected by a peroxidase-conjugated goat anti-mouse IgG (GE Healthcare Ltd., Hatfield, UK) using tetramethyl benzidine as substrate. After 30 min at room temperature, the enzymatic reaction was stopped by adding 50 μL of 1 M sulphuric acid/well. Washing steps were done with 200 μL/well of PBS containing 0.05% Tween-20 in an automatic washer. 

### 2.8. CTL Assay

Splenocytes from inoculated mice were cultured for 4 days in RPMI 20% FCS in the presence of 5 μg/mL of the above-described E7 or control peptides. After 4 days, the CD8^+^ cell fraction was isolated by positive immunomagnetic selection (Miltenyi Biotec.), and maintained overnight in RMPI 20% FCS in the presence of 10 U/mL of recombinant IL-2. After 16 h, EL-4 cells, previously labeled with carboxyfluorescein succinimidyl ester (CFSE, Invitrogen) and treated overnight with either E7 or control peptides, were co-cultured with CD8^+^ mouse splenocytes at different cell ratios (*i.e.*, from 1:100 to 1:2) in 200 μL of RPMI 20% in U-bottom 96-well plates. After additional 6 h, EL-4 cell mortality was scored by FACS analysis soon after the addition of 7-AAD at a final concentration of 1 μg/mL.

### 2.9. Anti-Tumor Effects of Nef^mut^/E7 Exosomes

C57 Bl/6 mice (5 per group) were inoculated following the previously reported schedule. Two weeks after the last exosome inoculum, mice were challenged with 10^5^ TC-1 tumor cells/mouse by s.c. injection. Tumor growth was monitored by visual inspection, palpation, and measurement of tumor nodule diameter.

Anti-tumor activity of Nef^mut^/E7 exosomes was also evaluated upon inoculation of mice already challenged with 2 × 10^5^ TC-1 cells. Exosome inoculations were performed 6, 11, and 19 days after tumor cell challenge only in mice which developed palpable tumors before the first immunization. At the end of the observation time, tumors were explanted and weighted. 

### 2.10. Statistical Analysis

When appropriate, data are presented as mean + standard deviation (SD). In some instances, the paired Student’s *t*-test was used and confirmed using the non-parametric Wilcoxon rank sum test. *p* < 0.05 was considered significant.

## 3. Results

### 3.1. Similar CD8^+^ T Cell Immune Responses Elicited by HPV-E7 Uploaded in Either Nef^mut^-Based Lentiviral VLPs or Exosomes

**Figure 1:**
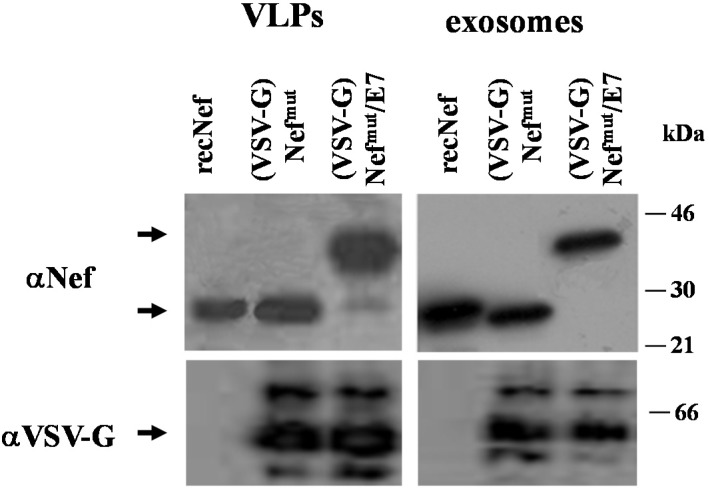

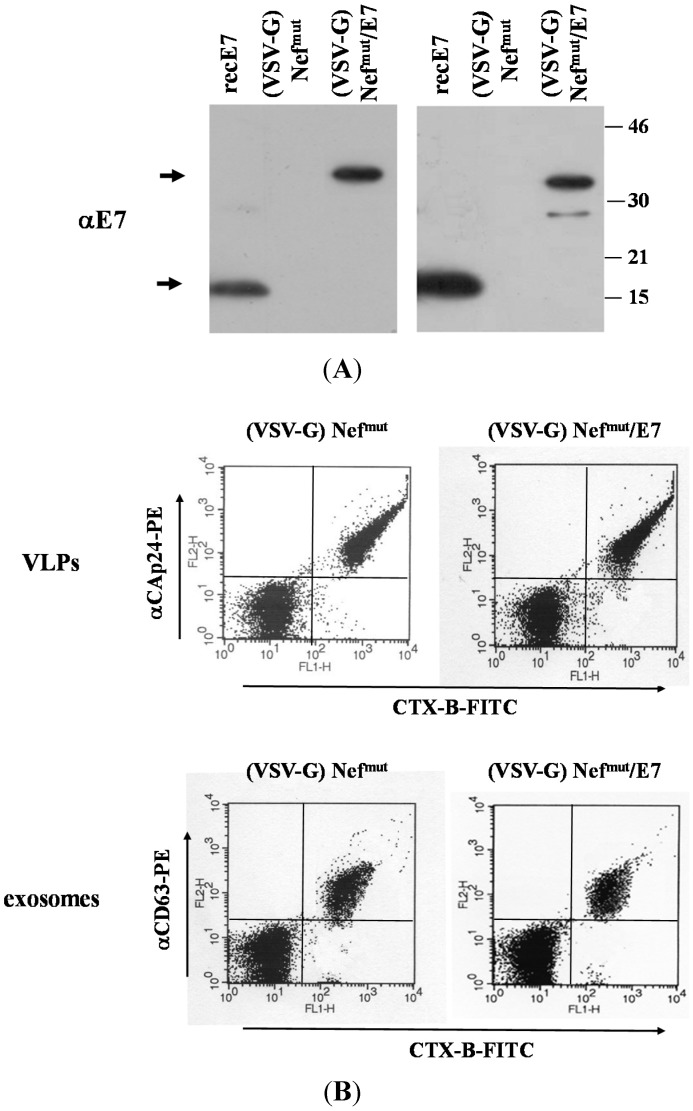
Molecular characterization of VLP and exosome preparations. (**A**) Western blot analysis of (VSV-G) VLPs and exosomes incorporating either Nef^mut^ or Nef^mut^/E7. In the left panels, the contents of both (VSV-G) Nef^mut^ and (VSV-G) Nef^mut^/E7 VLPs as revealed by anti-Nef, anti-VSV-G, and anti-HPV-E7 Abs are shown. The same Abs were used to detect the contents of (VSV-G) Nef^mut^ and (VSV-G) Nef^mut^/E7 exosomes (right panels). As control, 100 ng of either recombinant Nef or E7 were loaded. Arrows indicate the relevant protein products. Molecular markers are given in kDa. Results are representative of five independent experiments; (**B**) FACS analysis of both VLPs and exosomes incorporating either Nef^mut^, or Nef^mut^/E7. Equivalent amounts of VLPs and exosomes were bound to surfactant-free white aldehyde/sulfate latex beads, and then assayed for the contents of GM1 and HIV-1 CAp24 (VLPs), or GM1 and CD63 (exosomes). Quadrants were set on the basis of the fluorescence of beads alone labeled with the respective ligands. Results shown in both panels are representative of four assays carried out on two different VLP and exosome preparations.

Retro- and lentiviral VLPs are flexible vehicles for foreign immunogens. However, major hindrances regarding safety and ease of production strongly limit their potential application in clinic. The identification of the Nef^mut^ allele having an extraordinary ability to incorporate into both lentiviral-basedVLPs and exosomes even when fused with heterologous proteins opened the possibility to compare the two nanovesicle types in terms of efficiency of immunogen delivery. To this end, preparations of lentiviral VLPs and exosomes incorporating either Nef^mut^ alone or the product of its fusion with HPV-E7 protein were obtained and characterized. Both nanoparticle preparations were decorated with the G protein from vesicular stomatitis virus (VSV-G) to improve the delivery of nanoparticle contents in the cytoplasm of APC, thus favoring cross-presentation. Figure 1A shows the Western blot analysis of 500 ng of CAp24 of VLPs and equivalent amounts of exosomes, *i.e.*, 100 μU of AchE activity. We estimated that the samples comprised about 5 × 10^9^ nanovesicles. As already shown for alternative Nef^mut^-based fusion products [22], Nef^mut^/E7 also incorporates in VLPs and exosomes with comparable efficiencies.

The nanoparticle preparations were identified by FACS analysis upon binding with aldehyde-sulfate latex beads in terms of contents of both monosialotetrahexosylganglioside (GM1) and HIV-1 CAp24in VLPs, and both GM1 and CD63 in exosomes (Figure 1B). 

**Figure 2:**
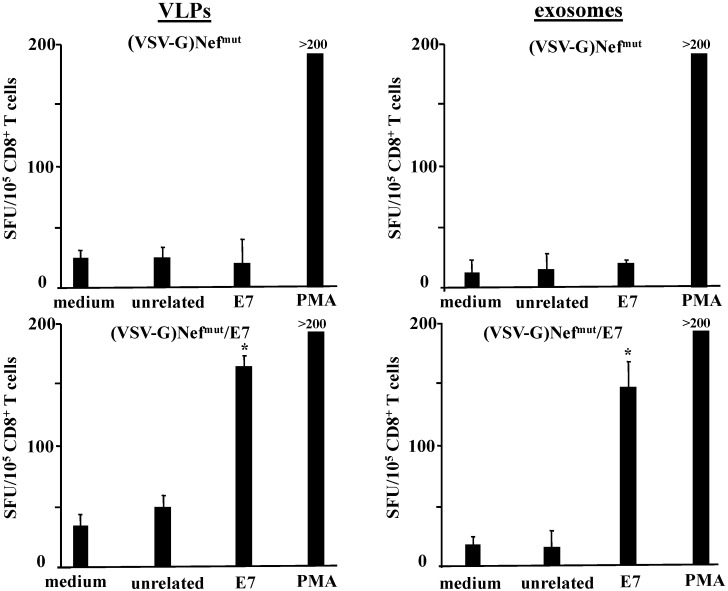
CD8^+^ T cell immune response in mice inoculated with (VSV-G)Nef^mut^-based VLPs or exosomes. C57 Bl/6 mice (five per group) were inoculated three times with VLPs or exosomes incorporating Nef^mut^/E7. As control, mice were also inoculated with equivalent amounts of both nanovesicle types incorporating Nef^mut^ alone. Splenocytes recovered from mice were incubated with or without 5 μg/mL of either unrelated or E7-specific peptides. Afterwards, cell activation extents were evaluated by IFN-γ Elispot assay carried out in triplicate with 10^5^ cells/well. As control, untreated cells were also incubated with 5 ng/mL of PMA and 500 ng/mL of ionomycin. Shown are the mean + SD number of IFN-γ spot-forming cells (SFU)/10^5^ cells. The results are representative of two independent experiments. * *p* < 0.05.

C57 Bl/6 mice were inoculated subcutaneously (s.c.) three times with volumes of VSV-G pseudotyped VLPs or exosomes containing 650 ng of immunogen, as determined by semi-quantitative Western blot analysis carried out using recombinant Nef as reference standard (not shown). Two weeks after the last inoculation, mouse splenocytes were isolated and cultured for 4 days in the presence or absence of either unrelated or HPV-E7-specific nonamers to selectively stimulate CD8^+^ T lymphocytes. Afterwards, 10^5^ cells were seeded in IFN-γ Elispot wells, and the number of spot-forming units (SFU) was scored 16 h later. Data reported in Figure 2 show that, in terms of induction of CD8^+^ T cell response, E7 uploaded in exosomes was as immunogenic as that delivered by VLPs. Of note, no anti-HPV E7 antibodies were found in sera from mice inoculated with either VLPs or exosomes (not shown). 

These results support the idea that Nef^mut^-based exosomes can be considered antigen carriers as efficient as Nef^mut^-based VLPs.

### 3.2. The Association of VSV-G to Exosomes Is Dispensable for Eliciting an Optimal Immune Response in Mice

The association of the fusogenic VSV-G envelope protein to exosomes was expected to favor presentation of cargo on Class I MHC. Consistently, we previously observed increased cross-presentation of foreign antigens uploaded in Nef^mut^-based exosomes when B-LCLs were challenged with VSV-G exosomes compared with non-pseudotyped exosomes [22]. However, inclusion of VSV-G or, expectedly, alternative pH-dependent fusogenic envelope proteins represents a major limitation in terms of scalable production of exosomes. Hence, we were interested in investigating the immunogenicity of Nef^mut^-based exosomes in the absence of envelope proteins. 

**Figure 3:**
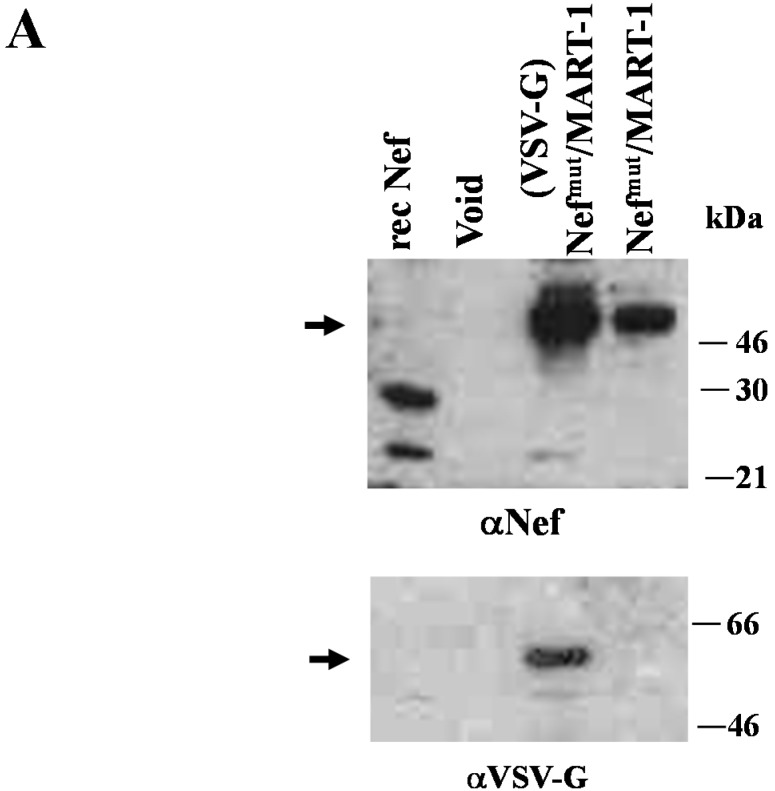

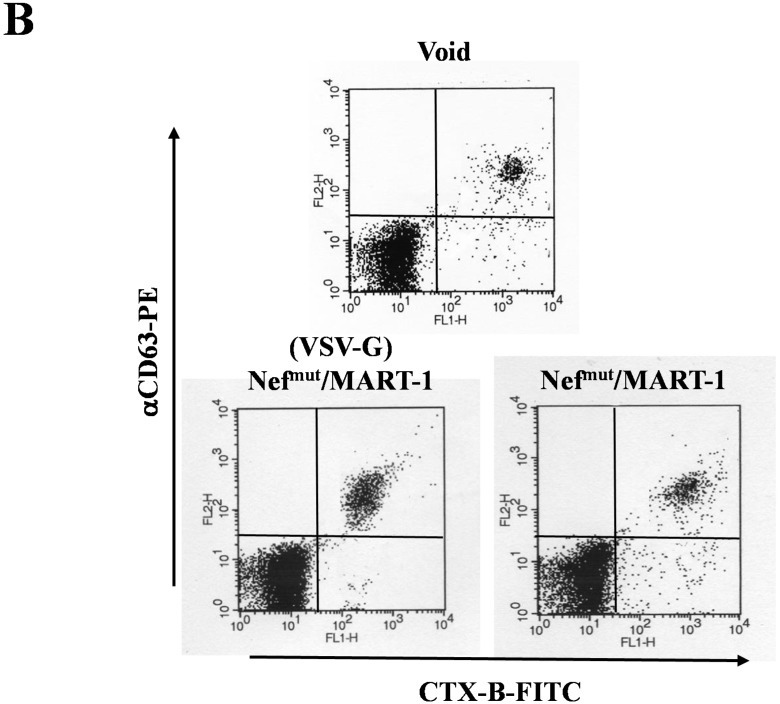
Molecular characterization of exosome preparations uploading Nef^mut^/MART-1. (**A**) Western blot analysis of 100 μU of AchE activity of exosomes associating Nef^mut^/MART-1 and pseudotyped or not with VSV-G. As control, exosomes from mock-transfected cells (void) and 100 ng of recombinant Nef were loaded. Shown are the results obtained upon incubation with either anti-Nef or anti-VSV-G Abs. The arrows indicate the relevant protein products. Molecular markers are given in kDa. Results are representative of two independent experiments; (**B**) FACS analysis of exosomes uploading Nef^mut^/MART-1 pseudotyped or not with VSV-G. Equivalent amounts of exosomes were bound to surfactant-free white aldehyde/sulfate latex beads, and then assayed for the contents of GM1 and CD63. Quadrants were set on the basis of the fluorescence of beads alone labeled with CTX-B and anti-CD63 mAb. Results shown in both panels are representative of three assays carried out on two different exosome preparations.

To this aim, we first reproduced the *in vitro* assays for cross-presentation of exosome-associated foreign antigen, however using monocyte-derived immature dendritic cells (iDCs) as APC instead of the previously tested B-LCLs [22]. This approach was pursued since iDCs represent an APC system more realistically recapitulating the events occurring upon *in vivo* exosome inoculation. The antigen cross-presentation assay was carried out by challenging HLA-A.02 iDCs with exosomes uploading either Nef^mut^ alone or the Nef^mut^/MART-1 fusion product. MART-1 (also known as Melan-A) is a human melanome-related tumor-associated antigen protein [36]. Its epitope at aa 27–35 represents an immunodominant domain restricted to HLA-A.02 Class I MHC [37]. Preparations of exosomes uploading Nef^mut^/MART-1 pseudotyped or not with VSV-G were characterized by Western blot analysis (Figure 3A), and in terms of both GM1 and CD63 contents (Figure 3B). As control, (VSV-G) exosomes uploading Nef^mut^ alone were used.

**Figure 4:**
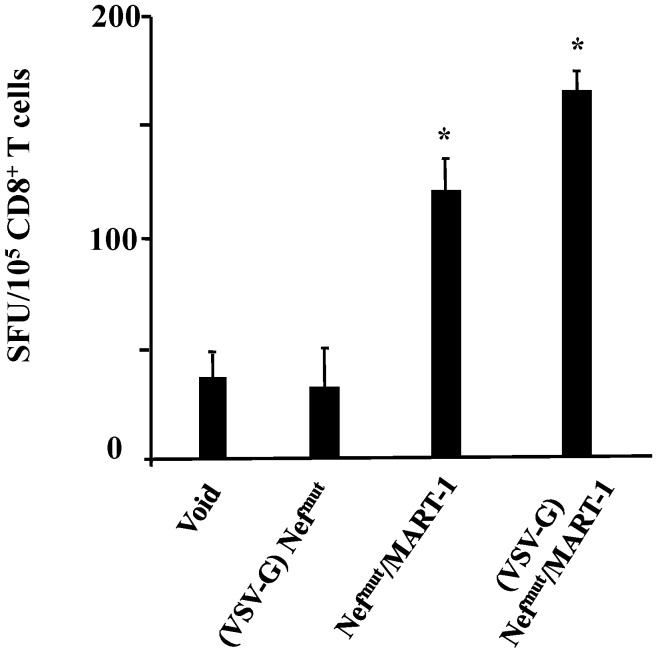
Cross-presentation of MART-1 delivered by exosomes. HLA-A.02 iDCs were challenged with 100 μU of AchE activity of exosomes associating Nef^mut^/MART-1 in the presence or not of VSV-G. As control, equivalent amounts of (VSV-G) Nef^mut^ exosomes or exosomes from mock-transfected cells (void) were used. After 4 h, the cells were put in co-culture overnight with a MART-1-specific, HLA-A.02-restricted CD8^+^ T cell line in IFN-γ Elispot microwells. Shown are the mean + SD number of SFU/10^5^ cells calculated from five independent experiments. * *p* < 0.05.

Equivalent amounts of either (VSV-G)Nef^mut^, (VSV-G)Nef^mut^/MART-1, or Nef^mut^/MART-1 exosomes were used to challenge iDCs. After 4 h, the cells were put in co-culture for 16 h in an IFN-γ Elispot 96-well plate with a CD8^+^ T cell clone recognizing the 27–35 epitope of MART-1. Figure 4 shows the increase of IFN-γ production in co-cultures comprising iDCs challenged with Nef^mut^/MART-1 exosomes, irrespective of the presence of VSV-G, compared with control conditions, *i.e.*, iDCs challenged with exosomes either from mock-transfected cells (Void), or uploading Nef^mut^ alone.

**Figure 5:**
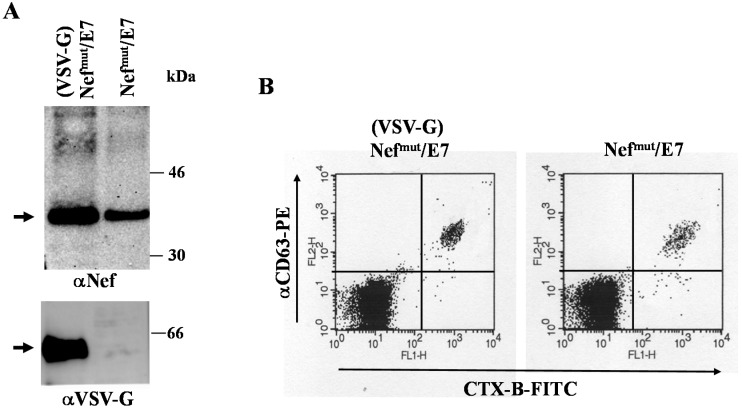
Molecular characterization of exosome preparations uploading Nef^mut^/E7 in the presence or not of VSV-G. (**A**) Western blot analysis of 100 μU of AchE activity of exosomes associating Nef^mut^/E7 in the presence or not of VSV-G. Shown are the results obtained upon incubation with either anti-Nef or anti-VSV-G Abs. Arrows indicate the relevant protein products. Molecular markers are given in kDa; (**B**) FACS analysis of exosomes uploading Nef^mut^/E7 in the presence or not of VSV-G. Exosomes were bound to surfactant-free white aldehyde/sulfate latex beads, and then assayed for the contents of GM1 and CD63. Quadrants were set on the basis of the fluorescence of beads alone labeled with CTX-B and anti-CD63 mAb. Results shown in both panels are representative of the assays performed on four different exosome preparations.

This result prompted us to compare the immunogenicity of exosomes associating or not VSV-G. Exosomes uploading Nef^mut^/HPV-E7 in the presence or not of VSV-G were prepared and characterized in terms of both antigen contents (Figure 5A) and membrane markers (Figure 5B). 

**Figure 6:**
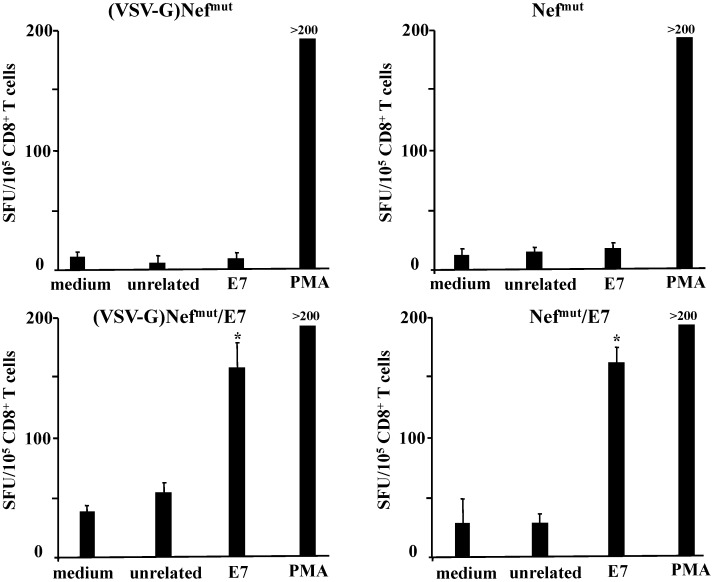
CD8^+^ T cell immune response in mice inoculated with Nef^mut^/E7 exosomes in the presence or not of VSV-G. C57 Bl/6 mice (5 per group) were inoculated three times with exosomes uploading either Nef^mut^ or Nef^mut^/E7, with or without VSV-G. Two weeks after the last inoculation, splenocytes were recovered and incubated 4 days with or without 5 μg/mL of either unrelated or the above-quoted E7 peptides. Afterwards, cell activation extents were evaluated by IFN-γ Elispot assay carried out in triplicate with 10^5^ cells/well. As control, untreated cells were also incubated with 5 ng/mL of PMA and 500 ng/mL of ionomycin. Shown are the mean + SD number of SFU/10^5^ cells. Results are representative of two independent experiments. * *p* < 0.05.

Afterwards, C57 Bl/6 mice were inoculated s.c. three times with exosomes carrying equivalent amounts of Nef^mut^/E7 in the presence or not of VSV-G. Two weeks after the last inoculation, mouse splenocytes were isolated and cultured for 4 days in the presence or absence of either unrelated or E7-specific peptides. Then, 10^5^ cells were seeded in IFN-γ ELISPOT wells, and the number of SFU was scored 16 h later. Data reported in Figure 6 show that E7 carried by exosomes without VSV-G was as immunogenic as that delivered by VSV-G pseudotyped exosomes. 

**Figure 7:**
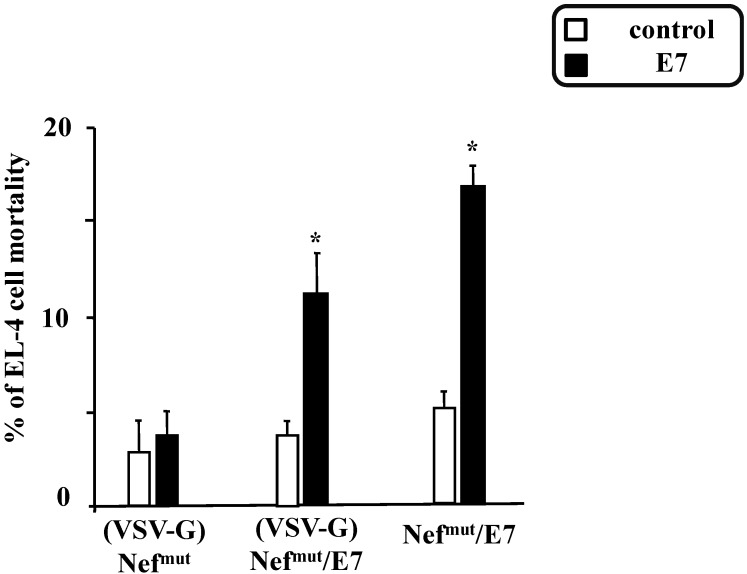
CTL assay carried out with CD8^+^ cells from mice inoculated with exosomes uploading Nef^mut^/E7 in the presence or not of VSV-G. Splenocytes from mice inoculated with either (VSV-G) Nef^mut^, (VSV-G)Nef^mut^/E7, or Nef^mut^/E7 exosomes were pooled and cultured for 4 days in the presence of either control peptides or the previously described E7 peptides. The CD8^+^ cell fraction was isolated and then co-cultivated for 6 h at different cell ratios (*i.e.*, from 100:1 to 2:1) with EL-4 cells previously labeled with CFSE and pre-treated with either unrelated or E7 peptides for 16 h. Finally, the cell mortality within the EL-4 cell population was scored by FACS analysis upon 7-AAD labeling. Shown are the results obtained with co-cultures carried out at a 20:1 cell ratio, and are representative of two independent experiments. * *p* < 0.05.

Consistently, similar anti-E7 CTL activity was detected in splenocytes inoculated with exosomes incorporating Nef^mut^/E7 pseudotyped or not with VSV-G (Figure 7).

### 3.3. Anti-Tumor Effects of Nef^mut^-Based Engineered Exosomes

Next, we investigated the potency of the immune response evoked by Nef^mut^-based exosomes in terms of anti-tumor activity. To this end, we used the well-characterized experimental tumor system consisting in the implantation in C57 Bl/6 mice of syngeneic TC-1 tumor cells which express HPV-E7 [26]. We evaluated the anti-tumor effects of the exosomes in both preventive and therapeutic settings.

**Figure 8:**
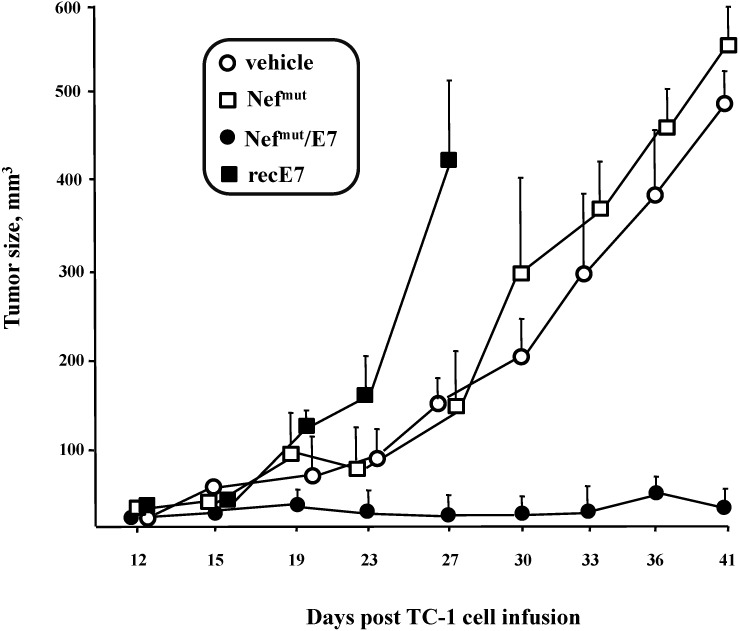
Growth of TC-1 tumor cells implanted in mice after inoculation of exosomes incorporating Nef^mut^/E7. C57 Bl/6 mice (5 for group) were inoculated three times at two-week intervals with exosomes incorporating Nef^mut^/E7 or, as control, with exosomes uploading Nef^mut^ alone. Mice were also inoculated with amounts of purified recombinant (rec) HPV-E7 protein equivalent to those associated with the exosome inocula. Two weeks after the last inoculation, mice were challenged with 10^5^ TC-1 cells, and tumor growth was monitored over time. Mice inoculated with recombinant E7 have been sacrificed at day 27 due to their heavily compromised health. Tumor size was calculated as (width × 2) × (length/2). Shown are the mean sizes + SD of tumors developed in mice within the different groups.

To investigate whether the immunity elicited by the Nef^mut^-based exosomes can counteract the growth of implanted TC-1 cells (preventive immunization), mice were inoculated three times with Nef^mut^/E7 exosomes, and, a week later, with 10^5^ TC-1 cells. As control, mice were inoculated with either vehicle, equivalent amounts of exosomes uploading Nef^mut^ alone, or amounts of recombinant E7 protein equivalent to those associated with each exosome inoculum. The growth of tumor cells was followed for 41 days. Tumor cells grew most efficiently in mice receiving recombinant E7 protein.Their sacrifice was anticipated in view of the rapid decay of their health conditions. Conversely, mice receiving Nef^mut^/E7 exosomes appeared efficiently protected by tumor cell challenge (Figure 8), indicating a strong anti-tumor efficiency of Nef^mut^/E7 exosomes when the immunogen was provided before tumor cell implantation.

**Figure 9:**
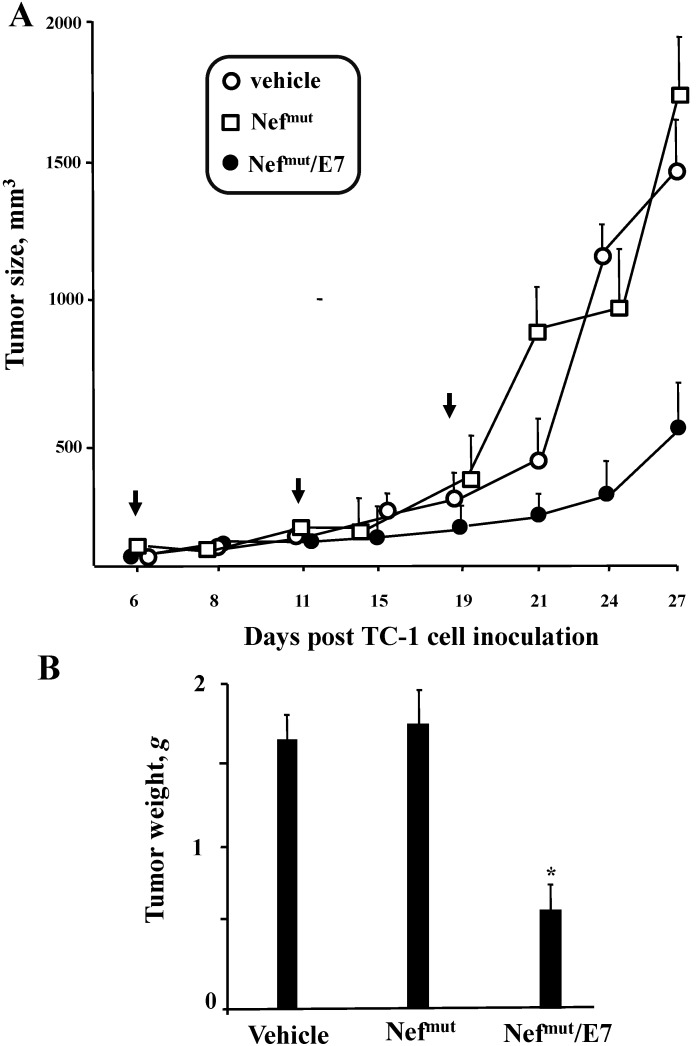
Anti-tumor effect of exosomes uploading Nef^mut^/E7 in mice implanted with TC-1 tumor cells. C57 Bl/6 mice (3 for group) were challenged with 2 × 10^5^ TC-1 cells and starting from 6 days later, when tumor mass was detectable by palpation, were inoculated 3 times with equivalent amounts of exosomes incorporating Nef^mut^/E7 or, as control, Nef^mut^ exosomes, or equal volumes of vehicle. (**A**) Determination of the tumor size over time calculatedas (width × 2) × (length/2). Shown are the mean sizes + SD of tumors developed in mice within the different groups. The times of exosome inoculation are indicated by arrows; (**B**) Measure of tumor weight. At the time of sacrifice, tumors were explanted and weighed. Shown are the mean weights + SD of tumors developed in mice within the different groups. Results shown in both panels are representative of two independent experiments.

To assay possible effects on already implanted tumor cells (therapeutic immunization), the engineered exosomes were applied in mice previously inoculated with 2 × 10^5^ TC-1 cells, and which developed a tumor mass detectable by palpation, *i.e.*, of about 2 mm of diameter within 6 days. The exosome inoculations were repeated three times. As reported in Figure 9, tumors grew slower in mice treated with Nef^mut^/E7 exosomes than in control conditions, *i.e.*, mice inoculated with vehicle or exosomes uploading Nef^mut^ alone.

We concluded that the inoculation in mice of engineered exosomes incorporating E7 elicits a CD8^+^ T cell response efficiently counteracting the tumor cell growth. 

## 4. Discussion

We aimed at establishing a novel way to produce CD8^+^ T cell immunogens against full protein antigens of choice. Our strategy was based on the use of engineered exosomes as immunogen carriers. Foreign antigens are uploaded in exosomes upon fusion at the *C*-terminus of a functionally defective HIV-1 Nef protein incorporating in exosomes at extremely high levels. In this paper, we report data supporting the proof-of-concept that, in this configuration, the foreign antigen is cross-presented and elicits a strong CTL response correlating with effective anti-tumor activity in both preventive and therapeutic settings.

Nanovesicles similar to exosomes can be released also through direct extrusion of plasma membrane [38]. Current protocols of purification cannot distinguish between endosome-produced nanovesicles and vesicles with similar size but extruding from cell membranes. The detection of tetraspannins, which associate with exosomes but not with plasma membrane nanovesicles, is considered an appropriate tool to identify true exosomes. On the other hand, GM1, *i.e.*, a structural component of lipid rafts which strongly binds the subunit B of cholera toxin (CTX-B), associates with both exosomes and nanovesicles arising from plasma membrane. The proportion of non-exosomal nanovesicles present in our exosome preparations can be deduced by the CD63/CTX-B double FACS analysis we reported in Figure 1B where the small populations of CTX-B+/CD63− vesicles likely account for the presence of plasma membrane nanovesicles which, however, appeared quantitatively negligible.

The immunogenicity of the foreign antigen incorporated in exosomes has been compared with that of the same antigen incorporated in HIV-1-based VLPs since: (i) VLPs are widely considered quite effective immunogens [39]; and (ii) both mechanisms and efficiency of Nef^mut^ uploading in VLPs and exosomes are basically similar. We previously observed that foreign antigens carried by Nef^mut^-based VLPs and exosomes are cross-presented with a similar efficiency [22]. Consistently, we here report that heterologous antigens incorporated in VLPs and exosomes elicit comparable CD8^+^ T cell-specific immunity. 

Antigen cross-presentation in DCs relies on two non-mutually exclusive mechanisms [40]. In the first one, referred to as “cytosolic,” the antigen transits from the endosomal compartment to cytosol. In the case of endocytosed vesicles, this passage can be greatly favored by pH-dependent envelope proteins like VSV-G. In cytosol, the antigen is degraded by proteasome and the resulting peptides are loaded on Class I MHC upon TAP-mediated translocation into endoplasmatic reticulum. The second mechanism is defined “vacuolar,” and includes the action of endolysosomal proteases which degrade both vesicles and associated proteins taken up by endocytosis. The resulting peptides are loaded into Class I MHC recycling at vesicular levels. We assumed that the results we obtained with exosomes deprived of VSV-G were the consequence of vacuolar cross-presentation activity of APCs ingesting the exosomes. 

Through the efficacy assays we carried out in mice we obtained the proof-of-concept that the CD8^+^ T cell immunity elicited by Nef^mut^/E7 exosomes correlates with the apparent destruction of target cells, which occurs in the absence of production of specific antibodies. On the contrary, inoculation of equivalent amounts of recombinant E7 had no effects in terms of CD8^+^ T cell-specific response, in the presence however of a well-detectable induction of specific antibodies. Hence, the association with exosomes strongly influences the type of adaptive immune response elicited against the antigen. This immune response can be potent enough to control the growth of tumor cells inoculated in mice before exosome immunization. In this case, the magnitude of the anti-tumor effect appeared comparable to that recently described in mice vaccinated with vaccinia virus-based vectors expressing E7 fused with calreticulin, *i.e.*, a protein strongly favoring Class I association of antigenic peptides [41].

Exosomes have been already a matter of consideration in human clinical trials. The major restriction in their use as vaccines has been identified in their overall limited immunogenicity. Since this aspect mainly depends on the amount of immunogen uploaded in exosomes, finding a reliable method to overload exosomes with the antigen(s) of choice would be a powerful way to strengthen their immune potency. With these premises, engineering exosomes exploiting the unique efficiency of exosome uploading of Nef^mut^ represents an original strategy whose potentialities in terms of both immunogenicity and anti-tumor efficacy have been disclosed by the results presented here. 

At present, the identification, production, and marketing of CTL-based vaccines are quite limited although there is a wide consensus about their potential usefulness against chronic infections and tumor diseases. Most commonly, efficient CTL immune responses can be achieved when the immunogen is either associated with or expressed by inactivated viral particles, viral vectors, or virus-like particles. In these cases, the presence of viral nucleic acids and proteins can represent a risk factor. Conversely, the use of exosomes virtually guarantees the absence of potentially dangerous viral material. In other instances, the combination of recombinant proteins with particulate adjuvants has been found effective in eliciting CTL immunity. Considering that in Nef^mut^-based exosomes the antigen resides inside the nanovesicle, neutralization by pre-existing or induced immunity, as can occur for both recombinant proteins and viral vaccines, is not expected to take place even in the case of repeated inoculations. All these features, together with the demonstrated flexibility in terms of incorporation of foreign antigens and ease of production, make Nef^mut^-based exosomes a convenient candidate for novel CTL vaccines. 
